# Ontology application and use at the ENCODE DCC

**DOI:** 10.1093/database/bav010

**Published:** 2015-03-16

**Authors:** Venkat S. Malladi, Drew T. Erickson, Nikhil R. Podduturi, Laurence D. Rowe, Esther T. Chan, Jean M. Davidson, Benjamin C. Hitz, Marcus Ho, Brian T. Lee, Stuart Miyasato, Gregory R. Roe, Matt Simison, Cricket A. Sloan, J. Seth Strattan, Forrest Tanaka, W. James Kent, J. Michael Cherry, Eurie L. Hong

**Affiliations:** ^1^Department of Genetics, Stanford University School of Medicine, Stanford, CA 94305, USA and ^2^Center for Biomolecular Science and Engineering, School of Engineering, University of California Santa Cruz, Santa Cruz, CA 95064, USA

## Abstract

The Encyclopedia of DNA elements (ENCODE) project is an ongoing collaborative effort to create a catalog of genomic annotations. To date, the project has generated over 4000 experiments across more than 350 cell lines and tissues using a wide array of experimental techniques to study the chromatin structure, regulatory network and transcriptional landscape of the *Homo sapiens* and *Mus musculus* genomes. All ENCODE experimental data, metadata and associated computational analyses are submitted to the ENCODE Data Coordination Center (DCC) for validation, tracking, storage and distribution to community resources and the scientific community. As the volume of data increases, the organization of experimental details becomes increasingly complicated and demands careful curation to identify related experiments. Here, we describe the ENCODE DCC’s use of ontologies to standardize experimental metadata. We discuss how ontologies, when used to annotate metadata, provide improved searching capabilities and facilitate the ability to find connections within a set of experiments. Additionally, we provide examples of how ontologies are used to annotate ENCODE metadata and how the annotations can be identified via ontology-driven searches at the ENCODE portal. As genomic datasets grow larger and more interconnected, standardization of metadata becomes increasingly vital to allow for exploration and comparison of data between different scientific projects.

**Database URL**: https://www.encodeproject.org/

## Introduction

The Encyclopedia of DNA Elements (ENCODE) project (https://www.encodeproject.org/) is an international consortium with a goal of annotating regions of the genome. The ENCODE project does this by identifying the regions that are bound by DNA- and RNA-binding proteins, investigating the chromatin structure, measuring transcriptional activity and measuring the extent of DNA methylation (1). The Data Coordination Center (DCC) is charged with validating, tracking, storing, visualizing and distributing these data files and their metadata to the scientific community (2). During the 6 years of the pilot and initial scale-up phase, the project surveyed the landscape of the *H. sapiens* and *M. musculus* genomes using over 20 high-throughput genomic assays in more than 350 different cell and tissue types, resulting in over 3000 datasets (3–6). In the current phase starting in 2012, the ENCODE project has added new genomic assays, a greater diversity of biological samples used in investigations, additional species (*D. melanogaster* and *C. elegans*) and new methods for validating and analyzing experimental data.

With the increase in the number of experiments, a greater level of organization of the data is demanded, so that results produced by the ENCODE consortium are readily accessible to the public. In the past, the DCC has utilized a controlled vocabulary to provide a consistent representation of experimental variables, known as metadata (7). However, controlled vocabularies are lists of words that lack connections between each other, making it difficult to identify related concepts. For example, the hepatic portal vein and the left lobe of a liver are both parts of a liver, but a list of controlled vocabularies does not capture this relationship. Therefore, it is difficult to retrieve all results related to the liver using a single search. To address this issue, the DCC, like many other biological databases, is using terms from the ontologies to describe the experimental metadata. By doing so, we continue to maintain a consistent representation of experimental variables and introduce new ways of searching and organizing the data (8, 9).

Here, we present the ontologies we use to annotate experimental metadata to provide improved searching capabilities across the data and to build a technical framework for ensuring the accuracy of the metadata. In this process, we are contributing to community efforts to improve the ontologies. By annotating the experimental metadata used to describe the ENCODE datasets with ontologies that are adopted by the scientific community, we facilitate the addition of continued data generation within the ENCODE consortium at the DCC, the utilization of ENCODE datasets by the larger genomics community and interoperability with other genomic databases.

## Why use ontologies?

A key goal of the ENCODE DCC, like other scientific resource projects, is to promote the discovery of relevant connections across experiments by providing structured and uniform descriptions of the data. The ENCODE DCC works with laboratories in the project to capture a set of metadata that includes what assay was performed, how the assay was performed, the biological sample that was investigated and any other reagents or experimental conditions that are essential to the interpretation of the data generated.

Ontologies are networks of controlled vocabulary terms (nodes) and relationships (edges) between the terms. Controlled vocabularies allow the same concept to be presented in a consistent way in different settings. For example, samples from the left lobe of a liver from multiple sources will consistently be described as ‘left lobe of liver’ and not just ‘left lobe’ or with a different capitalization. In addition, each term in the ontology can have one or more relationships to more general terms (often called ‘parent’ terms) and one or more specific terms (often called ‘child’ terms). This structure constitutes the current representation of knowledge in a given domain and forms a complex hierarchy, known as a directed acyclic graph (DAG), where any given node may have multiple children and/or parents (10, 11). These well-defined relationships can be used to satisfy general and specific queries of the data.

Therefore, a term in an ontology that is used to describe the metadata of an experiment allows it to be related to parent terms along the ontology graph. This association of the term to an experiment places that experiment in a greater context and allows it to be found using more general parent terms. As metadata from other experiments are annotated to terms in an ontology, those experiments become more accessible.

## Ontologies used for metadata annotations

**Figure 1.**
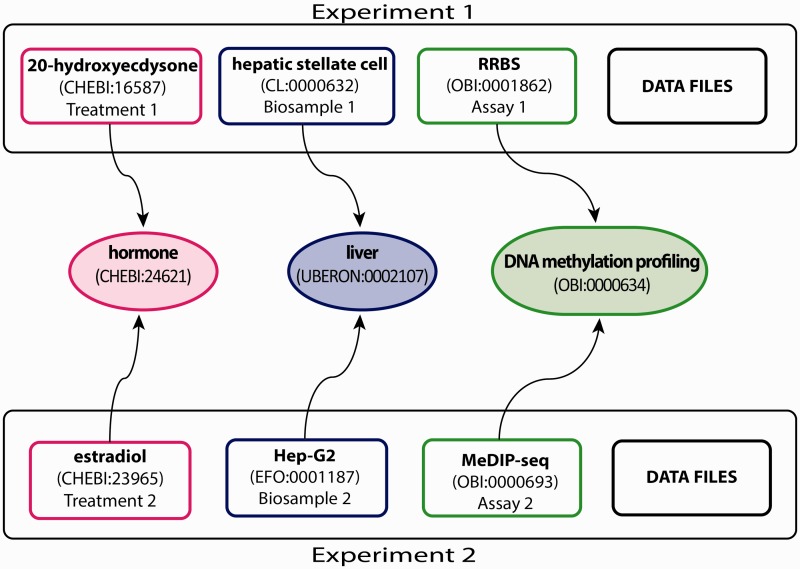
Experimental metadata annotated with appropriate ontology terms. This example, showing a subset of the full breadth of metadata annotated for an ENCODE experiment, emphasizes the annotation of three experimental metadata categories (treatment, biosample and assay) in two experiments. Treatments have been annotated to ChEBI (e.g. 20-hydroxyecdysone—CHEBI:16587 and estradiol—CHEBI:23965). Biosamples have been annotated to one of three ontologies: Uberon, CL and EFO (e.g. hepatic stellate cell—CL:0000632 and Hep-G2—EFO:0001187). Assays have been annotated to OBI (e.g. RRBS—OBI:0001862 and MeDIP-seq—OBI:000693). The terms in the middle are parent terms found in the ontology that provide a more general context and can be used to find these experiments (i.e. biological role for treatments, organ for biosamples, assay category for experimental assays). Each annotated term in the experiment maps, through relationships in the ontology, to the middle terms.

The ENCODE DCC organizes metadata related to the experimental process into several major categories that include donors, biosamples, treatments, constructs, libraries, antibodies, replicates and data files (V. S. Malladi, L. D. Rowe, E. L. Hong, *et al*., in preparation; see https://github.com/ENCODE-DCC/encoded/tree/mas ter/src/encoded/schemas for complete data model). We currently annotate three of these categories, a small subset of the metadata collected for an assay, using ontologies that provide the most additional information to the larger community (Figure 1). As illustrated in Figure 1, the following three categories of metadata are annotated with ontology terms:

1. The biological sample serving as input (i.e. biosample; e.g. hepatic stellate cell—CL:0000632 and Hep-G2—EFO:0001187 in Figure 1).
2. The reagents and conditions applied to the biological input (i.e. treatment; e.g. 20-hydroxyecdysone—CHEBI:16587 and estradiol—CHEBI:23965 in Figure 1).
3. The set of methods and conditions to survey the biological input (i.e. experimental assay; e.g. RRBS and MeDIP-seq in Figure 1). Annotating these metadata with ontologies provides alternate ways to search for these experiments (discussed below and Figure 1).

A single ontology is typically focused on modeling a specific set of relationships, such as the set of tissues and cell types that are part of an organ, the structural classes that are found in a chemical compound or key steps that comprise an experiment. Therefore, multiple ontologies have been selected to describe the three categories of experimental metadata listed above.

### Biosamples: Uberon, CL, EFO

No single ontology covers the scope of the biosamples used by the ENCODE project. Therefore, we categorized biosamples into seven types: (i) tissue, (ii) whole organism, (iii) primary cell, (iv) immortalized cell line, (v) *in vitro* differentiated cell, (vi) induced pluripotent stem cell and (vii) stem cell.

**Figure 2.**
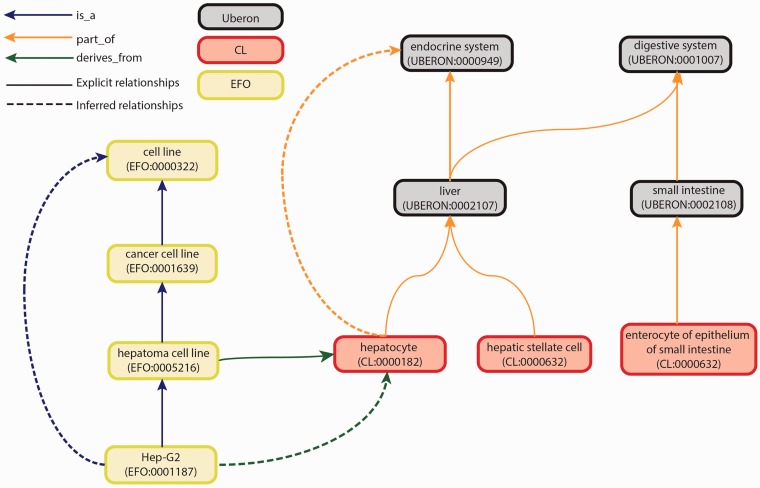
Graph view of integration of Uberon, CL and EFO. The graph view shows some of the relationship types and paths that can be traversed from child to parent terms. These relationships are either explicit or inferred. Explicit relationships are connections that are defined between two terms in the ontology. The integration of the three ontologies uses three relationships: is_a, part_of and derives_from. The is_a relationship indicates that one entity is a subtype of another entity (e.g. Hep-G2—EFO:0001187 is a type of hepatoma cell line—EFO:0005216). The part_of relationship indicates a part-whole relationship, such that an child term is fully and always contained within the parent term (e.g. all hepatocytes—CL:0000182 are found in the liver—UBERON:0002107). The derives_from relationship indicates that the child term succeeds parent term over some temporal divide, such that at least a significant biological portion is inherited (e.g. hepatoma cell lines—EFO:0005216 are cancerous hepatocyte cells—CL:0000182). Inferred relationships are connections between two terms that are transitively reasoned via the explicit relationships. Transitive relationships remain true across multiple links of the relationships. For example, as the Hep-G2 cell line is a type of hepatoma cell line that is derived from hepatocytes, an inferred relationship can be made that the Hep-G2 cell line is also derived from the hepatocytes.

We then selected three ontologies to cover these categories: Uber anatomy ontology (Uberon), Cell Ontology (CL) and Experimental Factor Ontology (EFO) (Figure 2).

To annotate biosamples in the tissue and whole organism categories, we use Uberon (http://uberon.org), which is an anatomical ontology that includes structural, functional and developmental relationships with emphasis on cross-species integration (11). Uberon focuses primarily on anatomy and will be used to cover biosamples that can easily be described by structure, location and are a heterogenous mixture of cells (e.g. liver—UBERON:0002107 and heart left ventricle—UBERON:0002084). Terms in Uberon include relevant cross-references to key model organism anatomy ontologies, such as the *Drosophila* gross anatomy (FBbt) and the *C. elegans* gross anatomy (WBbt) (12, 13).

For biosamples that are primary cells or stem cells, we use CL (http://cellontology.org) for annotation (14). CL details individual cell types and so is used for homogeneous mixtures of cells that have been separated from their original structure but do not contain genetic changes that would alter their biology from the ontology description (e.g. hepatic stellate cell—CL:0000632, mesenchymal stem cell of the bone marrow—CL:0002540).

For biosamples that do not directly correspond to an anatomical structure or physiological cell type, we use EFO (http://www.ebi.ac.uk/efo). EFO covers biosamples that have been subjected to exogenous alterations in their biology or defy endogenous classification by their heterogeneity. This includes experimentally derived samples, heterogeneous cell populations derived from cultures and other biological components commonly used in experiments that do not have a singular anatomical term (15). In addition, terms in EFO can be related to a specific disease. Immortalized cell lines are annotated using EFO, as well as induced pluripotent stem cells and established stem cell lines (e.g. K562—EFO:0002067, induced pluripotent stem cell—EFO:0004905 and H1-hESC—EFO:0003042). In addition, we have decided to annotate the intended product of *in vitro* differentiations rather than capturing individual combinations of treatment and biosample in the ontologies. As a result, this category of biosamples can be annotated to either CL or EFO following the rules described above.

These three ontologies were selected because relationships exists between these ontologies, which provide links between an organ and a primary cell that is part of an organ and the anatomical source of an immortalized cell line. These integrated ontologies allow us to identify both developmental lineage and anatomical location of a given cell population. Uberon integrates with CL to provide details of the development and differentiation relationships of primary cells (11, 14). Additionally, EFO has integrated with Uberon and CL to identify the anatomical lineage of a given experimentally derived cell population, if relevant (Figures 1 and 2).

### Treatments: ChEBI

Treatments that can be applied to a given biosample may be chemical or biological. The Chemical Entities of Biological Interest Ontology (ChEBI, http://www.ebi.ac.uk/chebi) details the relationships of chemical compounds used in biological assays (e.g. 17beta-estradiol—CHEBI:16469 and dimethyl sulfoxide—CHEBI:28262), so both chemical derivatives and biological roles can be connected (16).

### Assays: OBI and SO

At the assay level, the Ontology for Biomedical Investigations (OBI, http://obi-ontology.org) comprises relationships for the design and implementation of biological investigations (e.g. ChIP-seq—OBI:0000716, RNA-seq—OBI:0001271, RRBS—OBI:0001862) (17). The Sequence Ontology (SO, http://www.sequenceontology.org) covers features that describe components of biological sequence (e.g. DNA—SO:0000352, RNA—SO:0000356, mRNA—SO:0000871 and microRNA—SO:0000276), which are investigated by an assay (18). Annotating metadata properties to these ontologies will standardize our representation of the objectives of a given experiment and relate that experiment to other assays with similar aims across various projects. For example, although the RNA population being sequenced may be polyadenylated-RNA or total RNA, both experiments will be annotated to the RNA-seq assay. The detail of the RNA population being sequenced is captured as a separate piece of experiment metadata.

## Making metadata annotations

Each annotation to an ontology term is manually reviewed to ensure accuracy. This process involves reviewing protocols, reagent vendor materials and primary literature to provide additional detail and verification for metadata of each category. Biocurators use this information to inform them on what term in the ontology accurately describes the metadata. Because ontologies need to be adjusted to accurately reflect new and developing scientific results as well as areas of science that are being actively investigated, making metadata annotations often involves interacting with different ontology development groups. If a term does not exist, the biocurators use the gathered information to provide guidance on how to improve the ontology. Below we describe the curation process of identifying the appropriate ontology term for annotation and guidelines for submitting a change to the ontology developers, so that the ontology developers can implement terms.

### Selecting the ontology term

To annotate to the most relevant ontology term, biocurators use software tools Protégé (http://protege.stanford.edu/), Bioportal (http://bioportal.bioontology.org) (19), Ontology Look Up Service (http://www.ebi.ac.uk/ontology-lookup/) and OLSVis (http://ols.wordvis.com/) (20, 21). These tools allow for exploration of the ontology in various ways and can improve the determination of a selected terms relevance to the category being annotated. The combination of metadata curation and ontology exploration directs biocurators to annotate a selected component to a specific ontology term. Biocurators search for the most specific possible term available in a relevant branch of the ontology. Before using an ontology term, the definition of the term and relationships of that term within the ontology are reviewed for consistency with the experimental variable being annotated. Missing ontology terms or terms that contain inconsistencies are reported to the ontology developers via public issue tracking systems. Issue trackers allow for the community to discuss the validity of new terms and determine if additional clarification is needed. The name of the new term, its new relationships and references identified during the curation process are submitted to the relevant ontologies using their designated format of issue submission (Uberon—https://github.com/obophenotype/uberon/issues, CL—http://sourceforge.net/p/obo/cell-ontology-cl-requests/, EFO—https://www.ebi.a c.uk/panda/jira/browse/FGPTO-730?jql=project%20%3D%20FGPTO, OBI—http://sourceforge.net/p/obi/obi-terms/).

### Checking ontology relationships

For an ontology term selected for use in an annotation, we investigate the existing relationships within the ontology and determine if relationships exist that could be traced back to an Uberon term. This is done to ensure that a term describing a primary cell type in CL could be related to the organ it can be found in or an immortalized cell line in EFO could be related to its source tissue. If these relationships across ontologies are already present, we verify their accuracy through references to primary literature. If they are not present, we again consult primary literature to determine the expected relationship to an Uberon term. Using the expected relationship as a guide, we investigate the branches starting at either the Uberon term or the selected ontology term, from CL or EFO and proceed across them to determine the missing relationship that would integrate the branches across ontology boundaries. We attempt to bridge the ontologies by adding a relationship between the most specific parent term possible in one ontology and the most generic term possible in the other ontology to maximize connectivity across the ontology boundaries. For example, rather than connect cell line GM12878 (EFO:0002784) directly to B cell (CL:0000236), we used a more general term B-cell-derived cell line (EFO:0001640) to extend the relationships to current and future B cell lines. For cases in which two ontology boundaries needed to be crossed, we applied similar logic to both boundaries. Once determined, the required relationships are reported to the relevant ontologies using their designated format of issue submission. Details and references for the requested additions are also submitted to ontology specific issue trackers.

### Extending the ontologies

Determining connections between ontologies is just one component of ontology development that is required to facilitate its usage. Many ontologies are community-based efforts that rely on submissions by users to help in their development. As new experiments are submitted to the ENCODE DCC, some will not have corresponding terms in their respective ontologies. To annotate these experiments, a new term must be created. This process involves primary research to collect the appropriate annotations for the term. This term must also be assigned the appropriate relationships in the ontology graph. We have already requested the addition of ∼200 new terms (174 biosamples, 22 assays) to the ontologies listed above. As of December 2014, approximately 20% of all terms used to annotate the seven biosample types are awaiting a new term in the ontology (81 out of a total of 411 unique biosample terms used for annotation). The 22 new term requests for assays represented about 50% of all assays performed by the ENCODE Project at the beginning of our annotation efforts. As assays are not added to the ENCODE project regularly, we anticipate only 1–2 new term request for an assay per year.

In addition to new terms, there can also be errors discovered within existing terms. These may be observed as annotation errors that conflict with our existing knowledge. Correcting these errors involves gathering the relevant citations to resolve the discrepancy. We may also find relationship errors, often when a biologically relevant connection between two terms is missing from the ontology. Investigating these errors may also involve review of other relevant ontologies to determine community consensus for these terms. The additional relationships are reported to the ontology using their issue tracker providing primary and secondary ontology relationships, if applicable.

## Searching the metadata

### Free text searching

**Figure 3.**
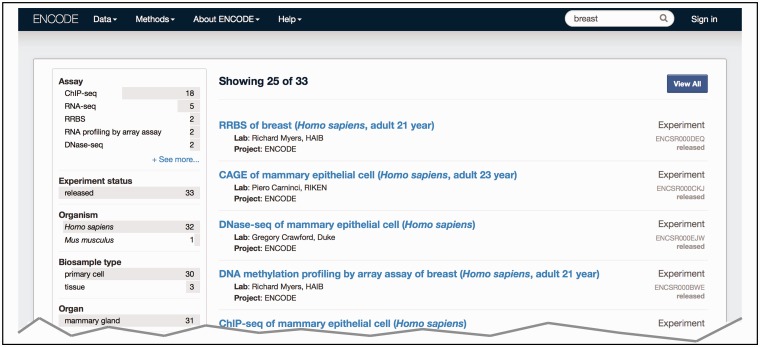
Search at the ENCODE portal (https://www.encodeproject.org/). In this example, a free text search is done for ‘breast’. The user selects ‘Experiment’ for the ‘Data Type’ facet. The interface returns a list of various experiments (right column) that have been conducted on biosamples that match the search term. The search uses the annotated ontological term for the biosample, synonyms found in the ontology or inferred relationships to the ontological term breast—UBERON:0000310.

The major benefit from using ontologies to annotate the experimental metadata is an increased ability of search queries to return related experiments (https://www.encodeproject.org/help/getting-started#search). Controlled vocabularies play a part in this by standardizing annotations to use the consensus terminologies that are most likely to be used as search terms and understood by users. In addition to the official term name, each term is associated with a range of synonyms that represent alternate phrasings or related concepts. We include the synonyms of the annotated term in the free text search. The inclusion of synonyms for the free text search allows controlled description of the metadata via the official term name but flexibility in the search term that is used via synonyms. For example, *H. sapiens* biosamples are annotated to the Uberon term ‘breast’ (UBERON:0000310), whereas *M. musculus* biosamples are annotated to the Uberon term ‘mammary tissue’ (UBERON:0001911) due to anatomical differences. However, searching the term ‘breast’ returns data for experiments using both *H. sapiens* breast tissue and *M. musculus* mammary tissue because the term ‘mammary tissue’ has the synonym of ‘lobe of breast’ (Figure 3)*.*

### Faceted searching

**Figure 4.**
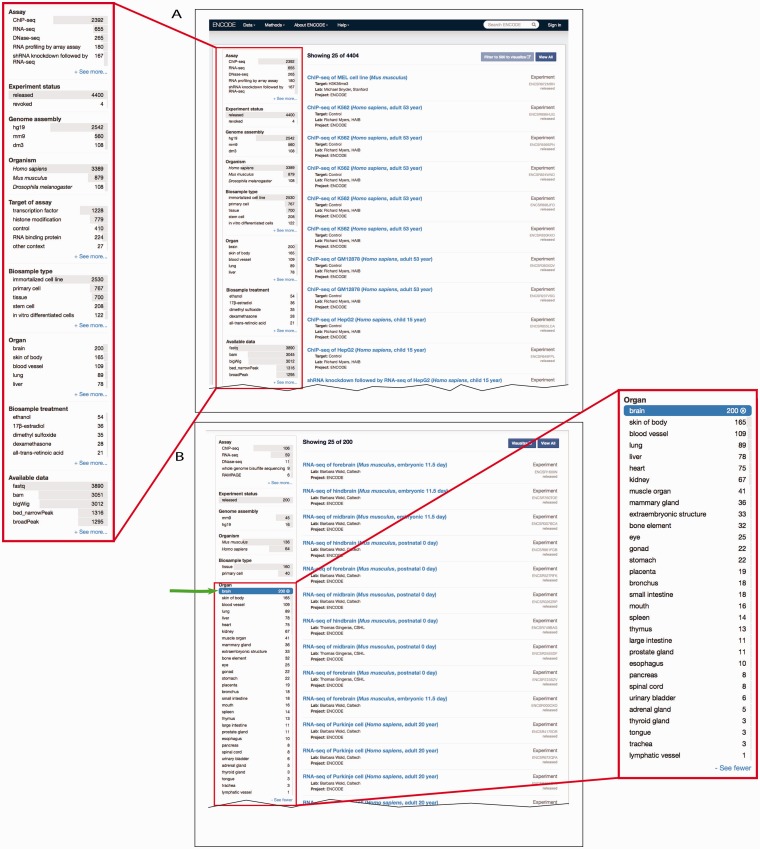
Filtering search results using facets. (**A**) A subset of facets for experimental assays is highlighted in the left column of the interface (https://www.encodeproject.org/search/?type=experiment). The ‘Assay’ facet displays the term name for annotations of an experiment to an OBI term id. ‘Experiment status’ indicates the state of the experiment record in the database. The ‘Organ’ facet represents the biosample slim described in the text describes the anatomical structure. The ‘Biosample treatment’ facet displays the term names for treatments, some of which are annotated to ChEBI. Lastly, the ‘Available data’ facet describes the data file types that are available for download from the ENCODE portal. (**B**) In this example, the user has expanded the ‘Organ’ facet and selected ‘brain’. To the right are all the available experiments on biological samples annotated to a specific term in Uberon, CL or EFO that slims to the parent term brain—UBERON:0000955.

Determining what to search for and how to search for it can be a daunting task. On the ENCODE portal, the metadata is easily available for search queries using a faceted searching system. Faceted search is a navigation technique that allows the user to explore the data by starting from a general query and subsequently filtering the results. Filtering is accomplished through components called facets, which are organizational units of information or metadata (22). We display these facets on the left hand side and allow users to apply multiple filters on the data (Figure 4A). For biosample-related facets, we have implemented a mechanism to filter the data using a subset of ontology terms, known as a slim, that represent broader categories of biosamples (Figure 4B).

### Creating facets via ontologies: biosample slims

A selected subset of ontology terms is commonly referred to as a slim. The Gene Ontology (GO) Consortium provides a classic example of a slim (http://geneontology.org/page/go-slim-and-subset-guide) (23). These are community created high-level subsets of GO terms that partition GO annotations into useful categories. These slims provide an overview of the specific terms that have been used to annotate a set of genes. The slim designation is determined by following an annotated term to its parent terms using relationships until either a parent term is in the list of slim terms or the parent term is a root term. As the ontologies we use are DAGs, there is more than one path from a term to the root allowing for the intersection of multiple slim terms for any given child term. A number of tools exist to map a set of specific annotations up to the corresponding set of GO slim terms, a process called slimming, including the web-based AmiGO (http://amigo1.geneontology.org/cgi-bin/amigo/slimmer) and command-line owl tools (https://code.google.com/p/owltools/). However, a limitation of these tools is that they require a known set of annotations. To accommodate our use of multiple ontologies and slims, we chose to write our own code (https://github.com/ENCODE-DCC/encoded/blob/master/src/encoded/commands/generate_ontology.py).

For biosamples, we have constructed a facet to describe the anatomical structure using terms found in Uberon (Figure 4B). These selected terms primarily represent major organs, including liver (UBERON:0002107), brain (UBERON:0000955) and heart (UBERON:0000948). Each selected slim term is listed in the facet when there are biosamples that slim to that term. For each annotated term in the database, we calculate all the facet terms that slim to it. These slims allow users to quickly subset the data by choosing one or more terms in a facet with no prior knowledge of the ontology terms or relationships. These slims also allow for a wider exposure and overview of the ENCODE data (https://www.encodeproject.org/help/getting-started#browse).

Faceted searching of all the data at the ENCODE portal involves a number of relevant metadata beyond what is contained within the ontologies we use, but the slims allow for the inclusion of the more complex relationships between experiments that are also biologically relevant (Figures 1 and 3). In constructing slims for this purpose, we provide easy access to metadata for a set of related biosamples when users do not know the full set of available terms to search.

### Metadata integrity using ontologies: assay slims

**Figure 5.**
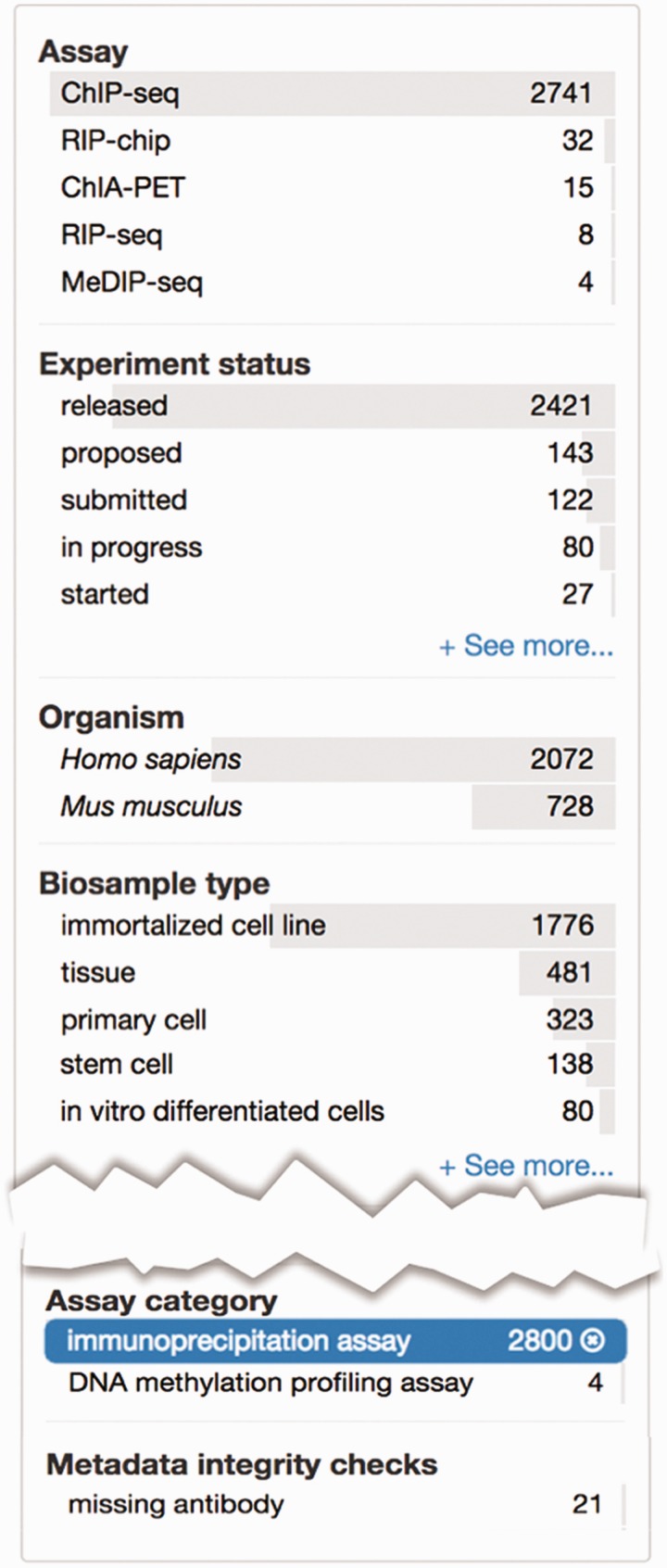
Metadata integrity using facets in the curation interface. This view highlights two additional facets: ‘Assay category’ and ‘Metadata integrity checks’ found on the curator interface for experimental assays. The selected term filters the ‘Assay’ facet, based on the assay slim described in the text, to only display a list of assays that can be categorized as immunoprecipitation assays. For these experiments, the ‘Metadata integrity checks’ facet can be used to filter for the experiments that are missing antibody information.

The relationship between terms in an ontology not only allows for a more complete representation of domain knowledge but also can be used to validate metadata annotated to these ontologies. As internal checks that are part of curation interfaces, we have created a facet using a subset of terms from OBI that organizes assays by major assay categories (Figure 5). The selected terms represent major types of categories that group related assays together. For example, the ‘immunoprecipitation assay’ includes ChIP-seq, iCLIP and ChIA-PET assays. This slim identifies assays which require an antibody to be listed in the metadata (Figure 5).

## Future directions

### Additional ontologies

Since we started annotating ENCODE metadata in late 2012, additional ontologies have been expanded or developed that may be used to capture additional categories of metadata. In addition, the assays performed by the ENCODE Consortium will continue to increase in diversity and complexity. Therefore, we will need to expand the ontologies that are used to appropriately capture these details in the metadata. For example, the Protein Ontology (PRO, http://pir.georgetown.edu/pro/pro.shtml) details proteins, their various modifications and relevant taxon information [e.g. transforming growth factor beta-1 (human)—PR:P01137] (24). Terms from PRO can be used to annotate biosample treatments that use protein reagents. With each new assay and analysis method comes new file formats. The EDAM Ontology (EDAM, http://edamontology.org) can be used to annotate the file format (e.g. FASTQ—format:1930, BAM—format:2572) as well as the data’s purpose (e.g. sequence alignment—data:0863) (25). The Biological Collections Ontology (https://github.com/tucotuco/bco) can be considered to annotate the method of sample collection and extraction (26). Like other ontologies, these ontologies can be used to improve searching, grouping and filtering of experiments to help identify the appropriate set of ENCODE experiments and to help ensure metadata accuracy.

With each additional ontology considered for annotation, the amount of manual work needed increases. To account for this, we plan on working with each of the ontology development groups, particularly for biosamples, to develop automated method of submitting new terms from our system to their respective tracking systems. For example, we have begun working with the developers of the CL to define rules that can help automate identifying and validating ontology relationships between new and existing ontology terms. For example, defining a rule that all new fibroblast terms from a specific organ needs to have ‘fibroblast’ (CL: 0000057) as a parent term will ensure those relationships are made. Although these rules can facilitate the addition of new terms to the ontology without manual review of the relationships, there will still be the need to ensure the correct ontology term is selected for the biosample that is being assayed

### Integrating datasets

As the number and variety of datasets increase, there is more need to compare results across different experiments. Faceted search is a powerful tool but is limited to the annotated data in a particular database. The wide distribution of data in different repositories requires the ability to search across these databases. The standardization and use of community maintained ontologies to annotate metadata allows for the ability to search using ontologies as links between databases, thereby creating a federation of databases.

Two such databases are the EBI Biosamples Database (http://www.ebi.ac.uk/biosamples/) and the NCBI Biosample Database (http://www.ncbi.nlm.nih.gov/biosample) (27, 28). These databases describe biological samples used in experimental assays recorded in various linked databases. The ENCODE DCC and EBI Biosamples Database both use EFO and ChEBI to annotate biological samples. The use of the same ontologies to annotate metadata in both databases allows for the community to search for experiments performed on the same or similar biological samples. The EBI provides an application programming interface to quickly query the database (http://www.ebi.ac.uk/rdf/services/biosamples/) (29). The ENCODE DCC has provided a similar interface, providing the ability to search using metadata terms (https://www.encodeproject.org/help/rest-api). The annotation of datasets using a common set of ontologies, coupled with search interfaces that allow programmatic integration of datasets from different resources, will facilitate the community’s ability to search for experiments without integrating the data into a single centralized database.

## Summary

The ENCODE DCC has organized the ENCODE project’s results to make them more available to the greater scientific community. To organize the data, the DCC has utilized a number of domain-specific ontologies to annotate experimental metadata. The integration of these ontologies provides the ability to quickly search all the ENCODE results using a variety of biological concepts. Further, the curation of all ENCODE metadata has led to enhancement of the ontologies and thus increasing their usefulness to the community. The wider adoption of these ontologies to annotate metadata is critical to allow interoperability between multiple databases.
